# Application of problem-based learning combined with three-dimensional visualization reconstruction technology in trauma orthopedics teaching and its impact on teaching satisfaction

**DOI:** 10.1186/s12909-025-08364-4

**Published:** 2025-12-07

**Authors:** Gang Xue, Siting Chen, JiaBing Xie, Min Yang, Zhengyu Wang

**Affiliations:** Orthopaedic department, The First Affiliated Hospital of Wannan Medical College, Yijishan Hospital, Zheshan West Road, Wuhu, China

**Keywords:** Trauma orthopaedics, Problem-Based learning, Three-dimensional visualization, Critical thinking, Teaching satisfaction

## Abstract

**Background:**

Trauma orthopaedics presents unique challenges in medical education due to complex fracture morphology and the limitations of traditional lecture-based approaches. Problem-Based Learning (PBL) emphasizes active participation and clinical reasoning, while three-dimensional visualization reconstruction technology (3D-VRT) enhances spatial understanding of anatomy and fracture patterns. However, the combined application of PBL and 3D-VRT in trauma orthopaedics education has not been fully evaluated.

**Methods:**

A single-blinded randomized controlled trial was conducted at the First Affiliated Hospital of Wannan Medical College, enrolling 50 clinical medical students between July 2023 and June 2024. Students were randomly assigned to the control group (traditional teaching, *n* = 25) or the experimental group (PBL combined with 3D-VRT, *n* = 25). Teaching effectiveness was assessed by written examinations, practical skill evaluations, a critical thinking disposition inventory, student evaluations of teaching methods, and a teaching satisfaction questionnaire.

**Results:**

Before the intervention, baseline performance showed no significant difference between the two groups. After the intervention, the experimental group demonstrated higher written examination scores (84.72 ± 2.39 vs. 79.52 ± 2.21, mean difference = 5.20, 95% CI: 3.62–6.78, *P* < 0.01) and practical operation scores (86.68 ± 2.22 vs. 79.24 ± 2.96, mean difference = 7.44, 95% CI: 5.82–9.06 *P* < 0.01) compared with the control group. Critical thinking ability scores were higher in the experimental group across all seven dimensions (total score 361.36 ± 11.46 vs. 256.96 ± 8.89, mean difference = 104.4, 95% CI: 95.1–113.7, *P* < 0.05). Student evaluations of teaching effectiveness (17.84 ± 1.12 vs. 10.72 ± 1.59, mean difference = 7.12, 95% CI: 5.51–8.73, *P* < 0.05) and overall teaching satisfaction (85.92 ± 4.34 vs. 74.96 ± 5.64, mean difference = 10.96, 95% CI: 8.24–13.68,*P* < 0.05) were also improved in the experimental group.

**Conclusion:**

The integration of PBL with 3D-VRT was associated with improved knowledge acquisition, practical skills, critical thinking ability, and teaching satisfaction among medical students in trauma orthopaedics education. This blended approach addresses the limitations of traditional teaching and offers a promising strategy for improving educational outcomes in trauma orthopaedics.

**Supplementary Information:**

The online version contains supplementary material available at 10.1186/s12909-025-08364-4.

## Introduction

Trauma orthopaedics is a highly specialized and technically demanding field due to the wide variability of fracture patterns, diverse anatomical sites of injury, and the multitude of available treatment options.Medical students and residents must acquire not only a solid understanding of orthopedic principles but also the ability to apply these principles flexibly across a range of complex clinical scenarios [1, 2].

One of the core challenges in teaching trauma orthopaedics lies in the inherently complex three-dimensional (3D) nature of fractures. These fractures are often presented through traditional two-dimensional (2D) imaging modalities such as plain radiographs, which can be insufficient for developing accurate spatial understanding [3]. As a result, learners frequently struggle with interpreting fracture morphology and visualizing anatomical relationships. Moreover, the diversity of trauma cases and the time-sensitive, high-pressure environment of emergency care further complicate training. Under such circumstances, theoretical instruction alone often fails to equip students with the practical decision-making and procedural skills needed in real-world settings [1].

Traditional didactic approaches—characterized by passive information delivery—contribute to low student engagement and limit opportunities for the development of hands-on surgical skills and clinical reasoning [4]. Without active participation, students may struggle to translate theoretical knowledge into clinical performance. These issues highlight the need for interactive and student-centered teaching strategies in trauma orthopaedics.

Problem-based learning (PBL) was first introduced in the late 1960 s at McMaster University in Canada, initially as a response to the limitations of traditional lecture-based medical curricula in cultivating clinical reasoning and self-directed learning skills [5]. Over the past decades, PBL has been widely adopted in medical education worldwide, demonstrating significant advantages in fostering active learning, enhancing problem-solving ability, and improving long-term knowledge retention [6, 7]. In surgical disciplines, particularly orthopaedics, PBL offers a case-centered approach that closely simulates real clinical decision-making processes and strengthens the link between theory and clinical application. This method emphasizes student autonomy, collaborative inquiry, and the active construction of knowledge [8]. In the context of trauma orthopaedics, where the complexity and urgency of clinical scenarios demand rapid and accurate decision-making, PBL fosters essential competencies such as clinical analysis, diagnostic reasoning, and collaborative communication [9]. This learner-centered strategy has been shown to enhance motivation, foster critical thinking, and improve retention of knowledge [10]. By simulating authentic clinical reasoning processes, PBL strengthens students’ ability to integrate theoretical knowledge into practice, making it an increasingly valuable pedagogical approach in trauma orthopaedic education [11].

Three-dimensional (3D) visualization technology has emerged as a powerful tool in trauma orthopaedics education by transforming CT or MRI data into high-resolution, interactive anatomical models. This approach allows dynamic exploration of anatomical structures and fracture morphology [12].Recent studies report that 3D visualization technology improves anatomical test performance and reduce surgical planning time [13–15]. Its interactive features—rotation, zooming, and transparency—support personalized and immersive learning.

Despite the proven strengths of PBL and the growing adoption of 3D visualization in medical education, studies examining their combined application in trauma orthopaedics remain limited. Given the unique demands of trauma orthopaedic training—where rapid decision-making, precise anatomical knowledge, and procedural planning are essential—innovative educational models are urgently needed to optimize learning outcomes. Therefore, this study aims to evaluate the effectiveness of combining PBL with 3D visualization technology in the teaching of trauma orthopaedics, with a focus on improving students’ learning performance, critical thinking ability, and overall satisfaction, while addressing the limitations of traditional teaching methods. We hypothesised that combining Problem-Based Learning (PBL) with Three-dimensional Visualization Reconstruction Technology (3D-VRT) would improve medical students’ knowledge acquisition, critical thinking ability, and overall teaching satisfaction compared with traditional lecture-based methods.

## Materials

A total of 50 clinical medical students who interned at the First Affiliated Hospital of Wannan Medical College between July 2023 and June 2024 were enrolled in this study. This was designed as a single-blinded randomized controlled trial. The students were randomly assigned to either the control group (*n* = 25) or the observation group (*n* = 25).Randomisation was performed using a computer-generated random number sequence created by an independent researcher who was not involved in the teaching or evaluation process. Allocation concealment was ensured by using sealed, opaque envelopes that were opened sequentially only after participant enrolment. The randomisation code was not disclosed to the instructors or evaluators until all data had been collected to maintain allocation blinding.The control group received traditional teaching methods, while the observation group was taught using a combined Problem-Based Learning (PBL) and Three-dimensional Visualization Reconstruction Technology approach. In the control group, there were 17 male and 8 female students, with a mean age of 25.08 ± 1.26 years. In the observation group, there were 15 male and 10 female students, with a mean age of 24.88 ± 0.67 years. Both groups received identical course content of trauma orthopaedics and teaching hours, delivered by the same instructor. All students had completed the theoretical trauma orthopaedics curriculum and departmental clinical rotations, and none had prior clinical work experience in trauma orthopaedics.

### Teaching methods

#### Experimental group

The experimental group adopted a Problem-Based Learning (PBL) approach integrated with three-dimensional (3D) visualization reconstruction technology, utilizing interactive anatomical models generated by software such as Mimics and 3D Slicer. Teaching was organized around typical clinical cases, and students were divided into small groups for discussion. They were encouraged to think independently and actively participate in problem-solving. Prior to each session, instructors posed relevant clinical questions, and students were required to search for information and summarize their findings.

High-resolution CT imaging data of fracture cases were retrieved from the hospital’s PACS system in DICOM format. These data were imported into Mimics (Materialise, Belgium), where bone tissue was segmented using threshold-based segmentation followed by region-growing and manual refinement to ensure accurate extraction of fracture fragments. The segmented masks were then converted into 3D surface models using the ‘Calculate 3D’ function, and smoothing algorithms were applied to improve the anatomical clarity of the models. The generated 3D models were exported in STL format and imported into 3D Slicer, where students could perform multi-angle rotation, zooming, slicing, transparency adjustment, and simulated fracture reduction using the ‘Transforms’ and ‘Model Clipping’ modules. This workflow follows reproducible and widely applied 3D reconstruction standards and is consistent with previously published applications of Mimics and 3D Slicer in surgical and medical education [16–19].

**Fig. 1:**
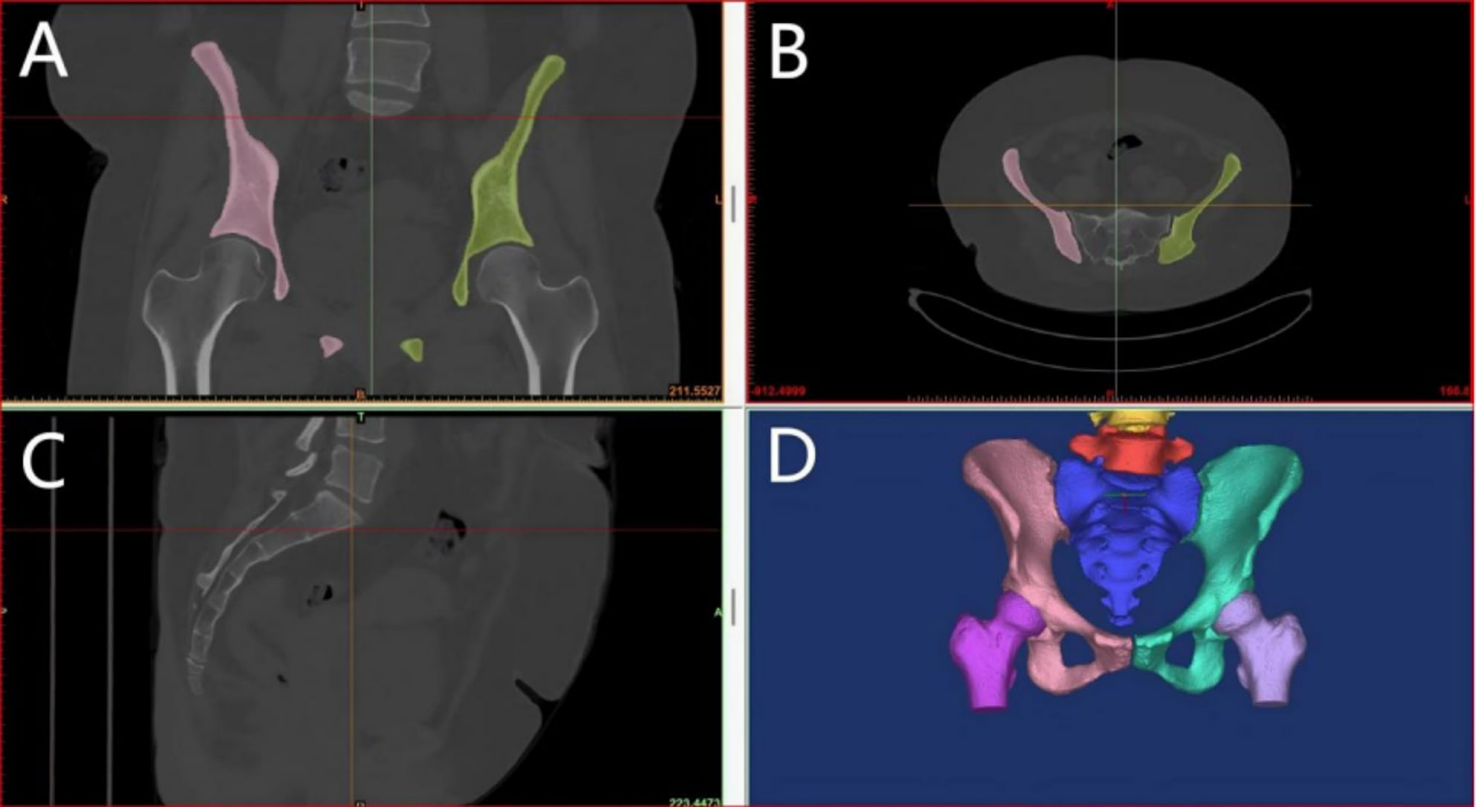
Three-dimensional visualization of pelvic CT data (**A**) Coronal view of CT reconstruction with highlighted bilateral iliac bones; (**B**) Axial CT reconstruction demonstrating bilateral iliac structures; (**C**) Sagittal CT reconstruction of the pelvis; (**D**) Three-dimensional reconstruction of the pelvis showing anatomical structures in different colors. These reconstructions, generated by Mimics, enabled students to rotate, zoom, and manipulate the models for better understanding of fracture morphology and spatial anatomy

**Fig. 2:**
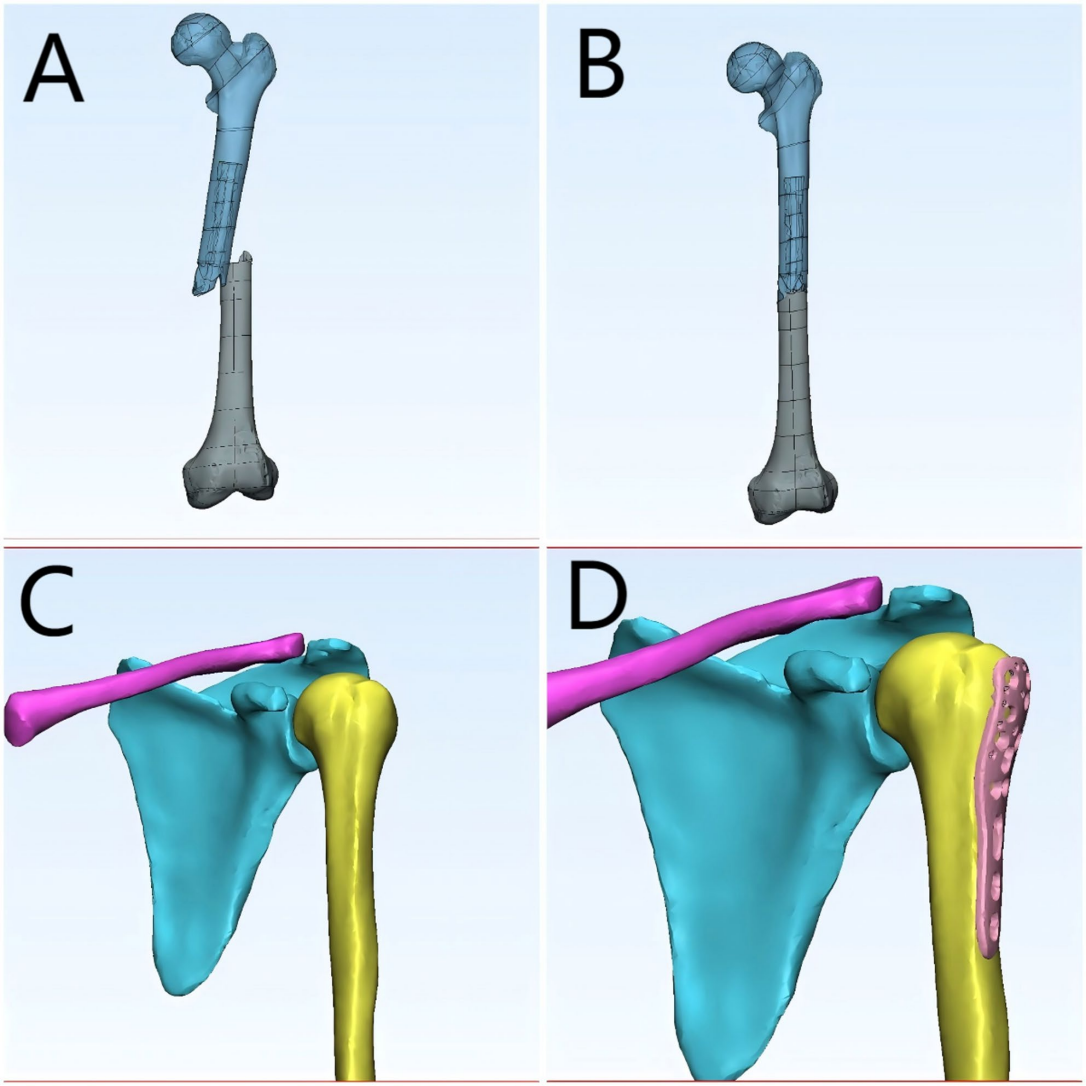
Application of 3D reconstruction models in fracture teaching of reduction and internal fixation (**A**–**B**) Segmented 3D models of the reduction of the femoral shaft fracture; (**C**–**D**) 3D simulation of internal fixation on the proximal humerus using a virtual plate. These models provided interactive tools for students to practice reduction, surgical planning, and fixation strategies in a problem-based learning (PBL) environment

**Fig. 3:**
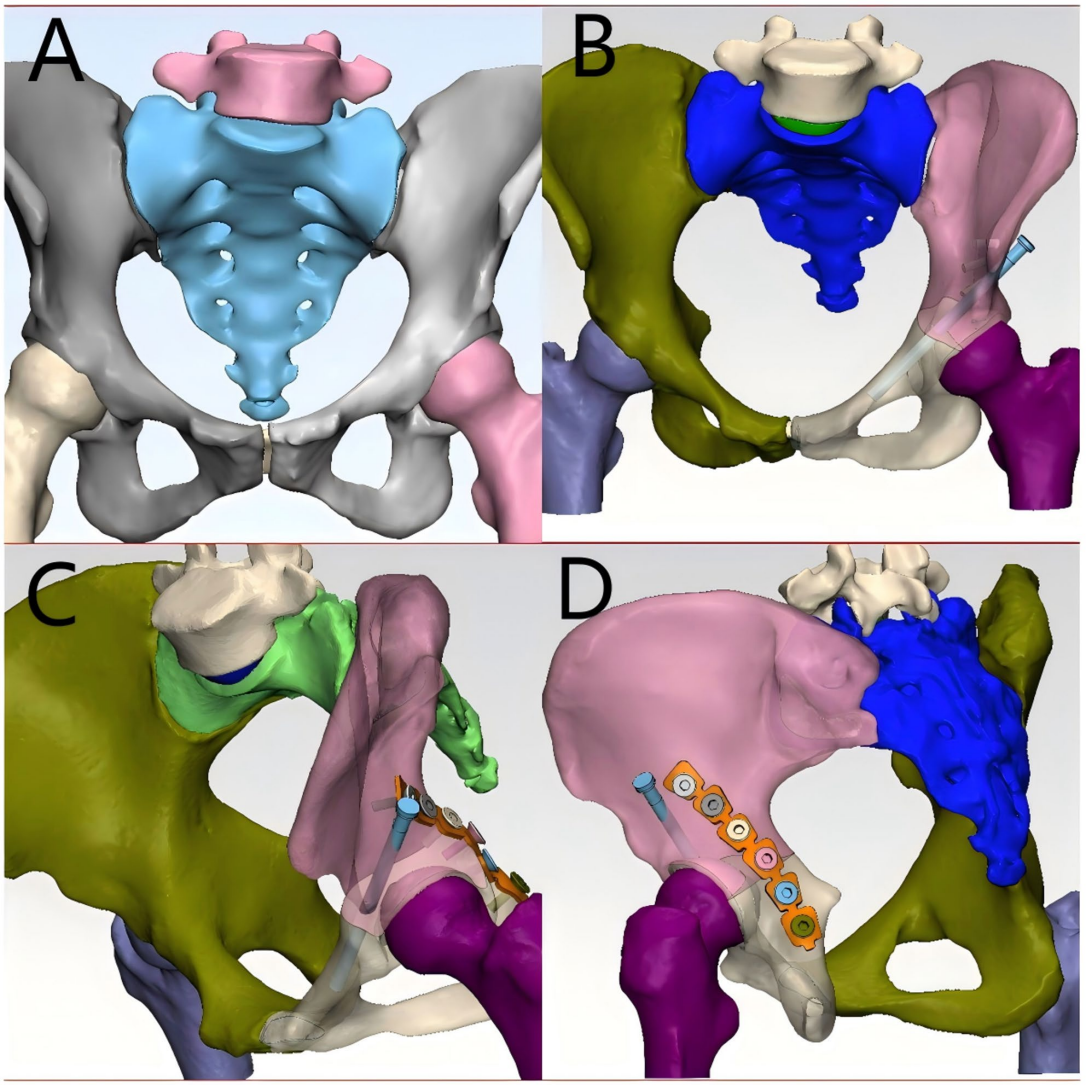
Three-dimensional visualization of acetabular fracture fixation (**A**) Pelvic 3D reconstruction highlighting the bony structures; (**B**) Acetabular fracture model with segmented anatomical components for preoperative planning; (**C**) Virtual simulation of fracture reduction and anterior acetabular column screw placement; (**D**) Application of virtual international fixation with plate and screws on the acetabulum This 3D reconstruction allowed students to visualize the fracture morphology, practice reduction techniques, and simulate internal fixation strategies, thereby enhancing their understanding of pelvic anatomy and surgical approaches in a problem-based learning (PBL) teaching environment

**Fig. 4:**
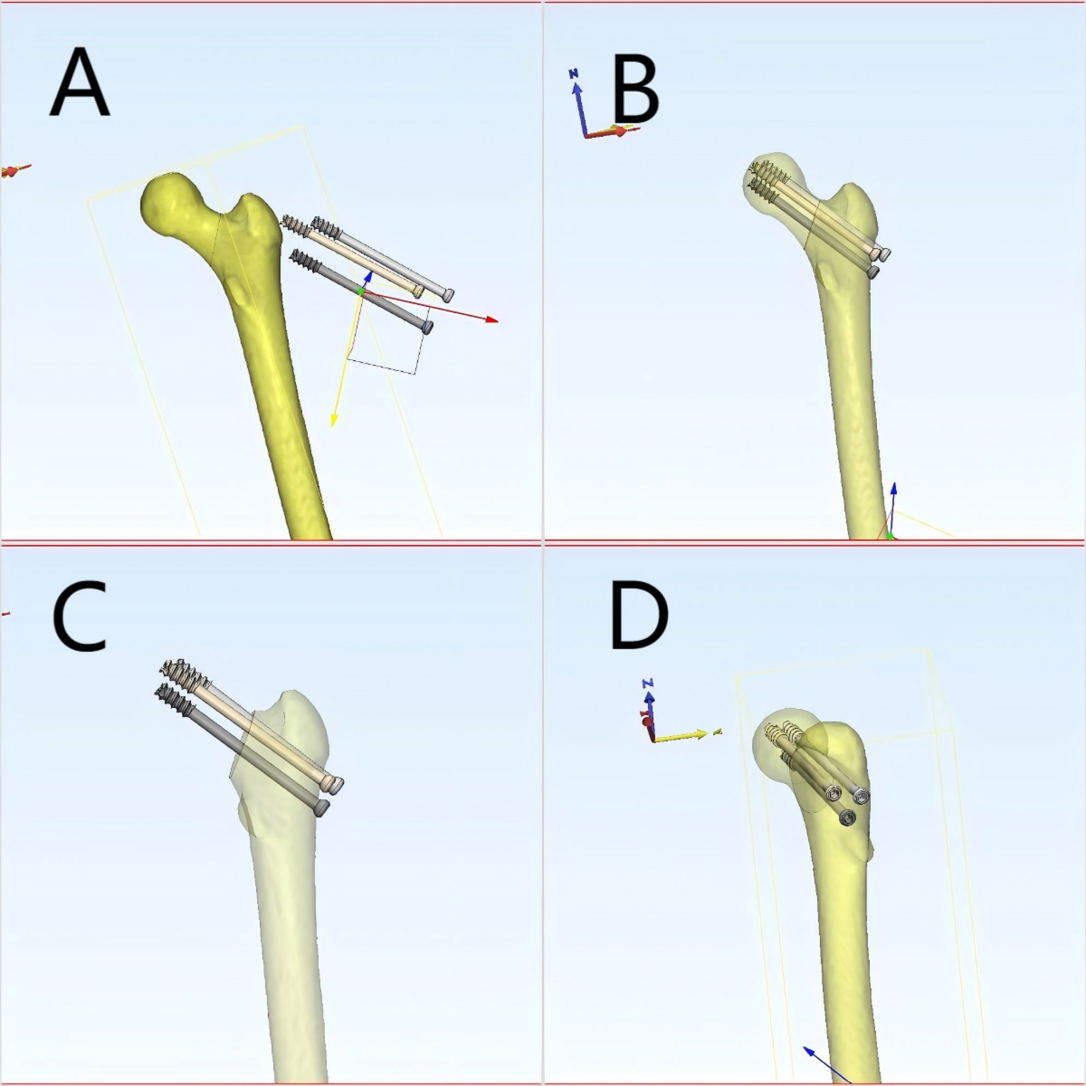
Three-dimensional visualization of femoral neck fracture fixation with cannulated screws. **A** Initial design of screw placement using Mimics software, showing the femoral shaft and proposed screw trajectories. **B** Detailed view of screw insertion angles and alignment within the femoral neck. **C** Simulated reduction and screw positioning after manipulation. **D** Final configuration of multiple cannulated screws, illustrating spatial arrangement and measurement of angles for optimal fixation.the 3D models facilitated a hands-on learning experience, enabling students to visualize and practice the internal fixation of femoral neck fractures with cannulated screws

During the teaching process, for complex fracture cases, CT data from actual patients were used to generate 3D visual models of the fracture site. Students were able to manipulate these models—zooming in and out, rotating, translating, measuring, slicing, and simulating reduction—to better understand the spatial structure and morphology of complex fractures. This interactive engagement helped deepen students’ understanding and memory of fracture classification and other difficult concepts. Students also used the 3D models to plan surgical approaches, thereby enhancing their clinical reasoning and teamwork(The teaching process is illustrated in Figs. 1, 2, 3 and 4). Instructors did not participate in group discussions directly but provided timely guidance when discussions deviated from the intended direction or when major disagreements occurred. Teachers assumed the roles of facilitators and recorders, guiding student elaboration, appropriately expanding related knowledge, and carefully recording each student’s performance. At the end of the session, instructors summarized the key discussion points, consolidated consensus, and addressed unresolved issues.

#### Control group

The control group followed a traditional teaching model. Students attended mini-lectures and participated in ward-based teaching rounds focused on typical clinical conditions. Mini-lectures were primarily delivered using PowerPoint presentations. During ward rounds, students presented patient cases, while instructors supplemented the history and performed physical examinations. The instructors explained the diagnostic and treatment processes of typical orthopedic conditions based on real cases. Students mainly listened passively, while instructors occasionally asked questions, and students responded accordingly. If students had questions, instructors answered them directly.

### Outcome measures and effectiveness evaluation

#### Clinical assessment

Both groups underwent standardized clinical evaluations, including written examinations and practical skill assessments. The written test covered theoretical knowledge (e.g., fracture classification, surgical principles) and clinical case analysis. Practical skill assessments focused on common procedures in trauma orthopedics, such as closed reduction and plaster immobilization for distal radius fractures. All exam questions were randomly selected, and both types of assessments were scored on a 100-point scale.

#### Student evaluation of teaching methods

A self-designed, anonymous orthopedic teaching evaluation questionnaire was distributed to both groups.(see Supplementary File 1).The questionnaire assessed five domains: knowledge acquisition, development of clinical reasoning, improvement of practical skills, enhancement of self-directed learning, and teamwork abilities. Each item was rated on a four-point scale: 1 (Not at all), 2 (General), 3 (Significant), and 4 (Very Significant), for a total maximum score of 20. Higher scores indicated better perceived teaching effectiveness.The internal consistency reliability of this questionnaire was confirmed by Cronbach’s α = 0.87, indicating good reliability. Content validity was established through expert review by three senior orthopaedic educators prior to implementation.The design of this self-developed questionnaire was informed by previously validated educational evaluation frameworks [1, 20, 21]. Its structure and scoring dimensions were adapted from established studies on medical education assessment and active learning outcomes.These references guided the domain selection and item phrasing to ensure theoretical consistency and content relevance.

#### Assessment of critical thinking ability

The Critical Thinking Disposition Inventory for medical students was employed to evaluate students’ critical thinking capabilities. The scale consists of seven dimensions: truth-seeking, open-mindedness, analytical ability, systematicity, critical thinking self-confidence, inquisitiveness, and cognitive maturity. Each dimension contains 10 items, rated using a 6-point Likert scale. The total score ranges from 70 to 420, with higher scores indicating stronger critical thinking dispositions.

#### Teaching satisfaction evaluation

An anonymous teaching satisfaction survey was conducted using a self-developed trauma orthopedics satisfaction questionnaire. (see Supplementary File 2).The questionnaire included six components: teaching effectiveness, learning interest, content quality, self-worth, humanistic competence, and teamwork. Each domain was scored out of 20, for a total of 100 points. A total score ≥ 90 was classified as “very satisfied,” 60–89 as “basically satisfied,” and < 60 as “dissatisfied.” Teaching satisfaction rate = (Very satisfied + Basically satisfied)/Total number of students × 100%.The questionnaire demonstrated good internal consistency (Cronbach’s α = 0.91). Content validity was reviewed by two medical education specialists and three clinical teaching experts to ensure appropriateness and clarity of the items.Similarly, this satisfaction questionnaire was developed with reference to established educational evaluation methodologies [22], incorporating validated constructs from prior studies in health professions education to enhance reproducibility and comparability.

### Statistical analysis

Statistical analysis: Analysis of variance (ANOVA) and chi-square tests were used to compare basic demographic information such as age and gender between the different fracture types. A post hoc power analysis was conducted based on the difference in written examination scores between the two groups (Cohen’s d = 1.02). The calculated statistical power was **0.91** at α = 0.05, indicating that the sample size of 25 students per group was sufficient to detect significant between-group differences.The Shapiro–Wilk test was used to assess the normality of data distributions, and Levene’s test was employed to examine the homogeneity of variances across groups.All clinical assessment scores, critical thinking scale scores, and teaching evaluation measures met the assumptions for parametric testing. For post-hoc comparisons following ANOVA, the Bonferroni correction was applied to control for multiple testing. In addition to *p*-values, effect sizes (Cohen’s d) were calculated and reported to provide information on the magnitude of differences between groups. All variables met the assumptions for parametric testing. Statistical significance was set at *p* ≤ 0.05. All data analysis was performed using SPSS v19 (IBM, Armonk, New York).

## Results

### Clinical assessment

**Fig. 5:**
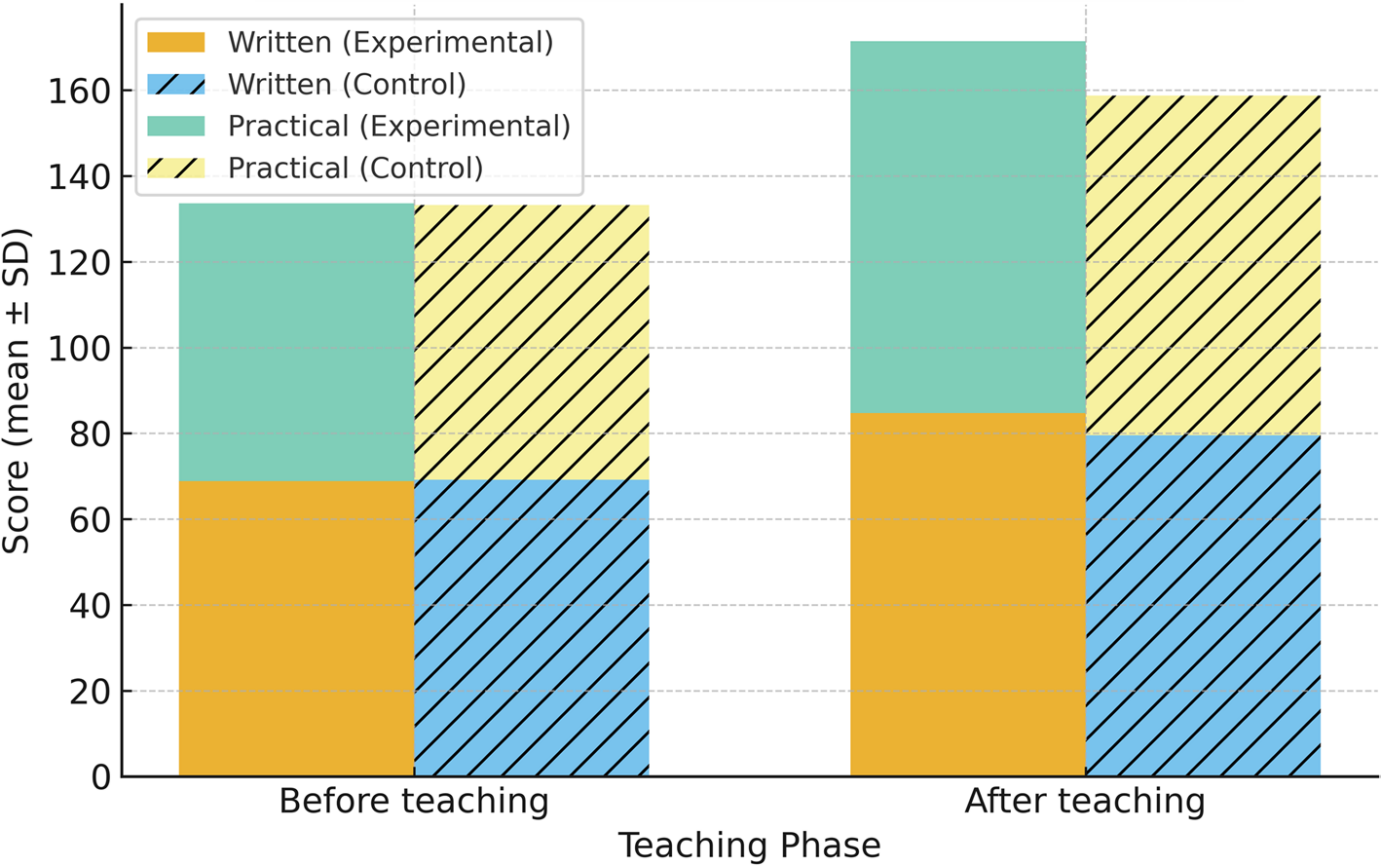
Comparison of written examination and practical operation scores between the experimental and control groups before and after teaching Data are presented as mean ± standard deviation. Error bars represent 95% confidence intervals. Statistical significance was determined by independent-samples t-test; *P* < 0.05 was considered significant

Before the intervention, there were no significant differences in written examination scores (experimental group: 68.84 ± 2.40; control group: 69.16 ± 1.80; *P* = 0.603) or practical operation scores (experimental group: 64.76 ± 2.07; control group: 64.08 ± 1.55; *P* = 0.203) between the two groups, indicating comparable baseline performance. After the intervention, the experimental group, which received Problem-Based Learning (PBL) combined with Three-dimensional Visualization Reconstruction Technology, demonstrated significantly higher scores compared to the control group. The written examination scores were 84.72 ± 2.39 for the experimental group versus 79.52 ± 2.21 for the control group (mean difference = 5.20, 95% CI: 3.62–6.78, *P* < 0.01). Similarly, practical operation scores were significantly higher in the experimental group (86.68 ± 2.22) compared to the control group (79.24 ± 2.96; mean difference = 7.44, 95% CI: 5.82–9.06, *P* < 0.01).(outlined in Table 1; Fig. 5).

**Table 1:** Comparison of written examination and practical operation scores between the experimental and control groups before and after teaching

Assessment	Timepoint	Experimental group (mean ± SD)	Control group (mean ± SD)	*P* value
Written examination (score)	Before teaching	68.84 ± 2.40	69.16 ± 1.80	0.603
Practical operation (score)	Before teaching	64.76 ± 2.07	64.08 ± 1.55	0.203
Written examination (score)	After teaching	84.72 ± 2.39	79.52 ± 2.21	< 0.01
Practical operation (score)	After teaching	86.68 ± 2.22	79.24 ± 2.96	< 0.01

### Critical thinking ability

**Fig. 6:**
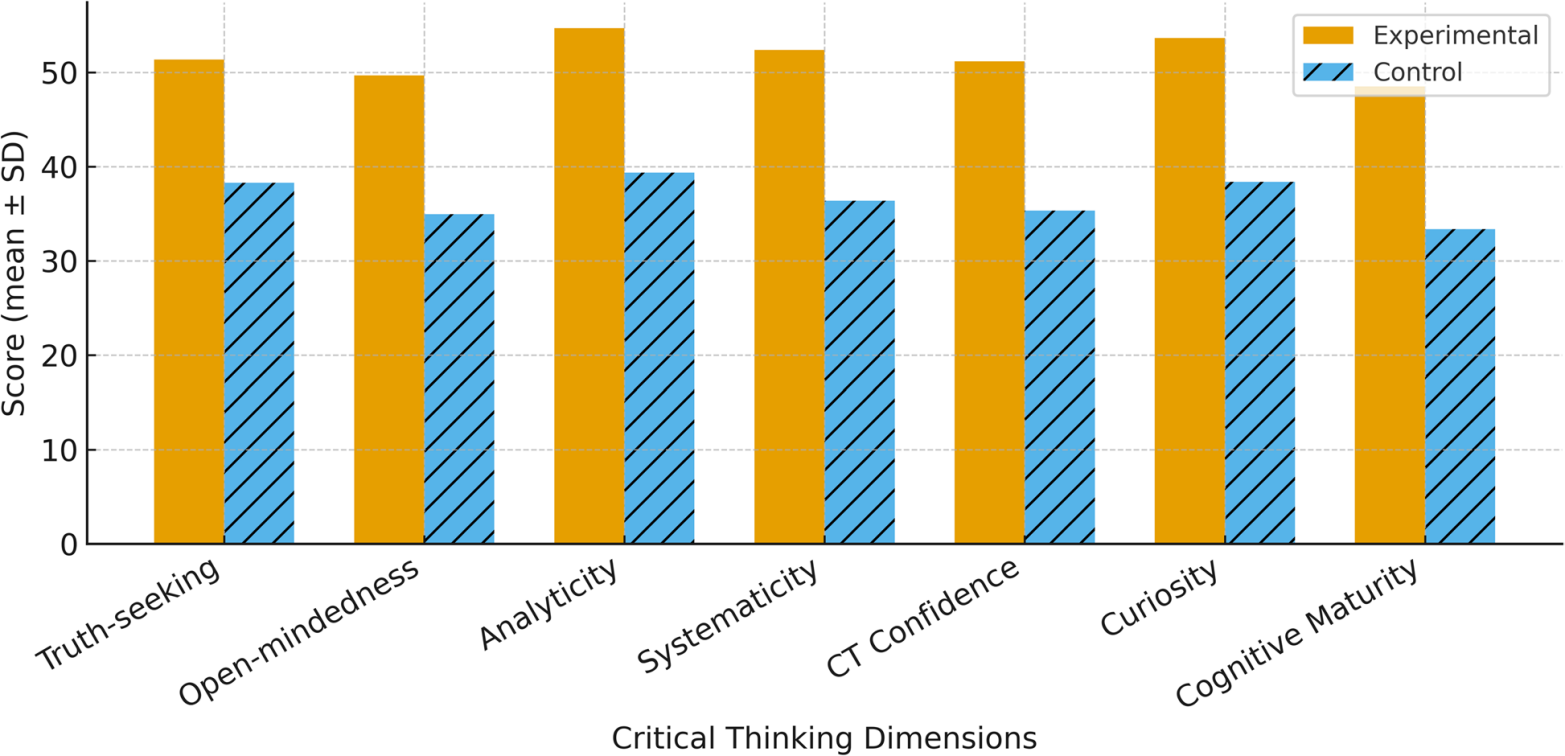
Comparison of critical thinking dimensions between the experimental and control groups after teaching Each bar represents the mean ± standard deviation for seven dimensions of the Critical Thinking Disposition Inventory. Error bars indicate 95% confidence intervals. All differences between groups were statistically significant (*P* < 0.05)

The experimental group exhibited significantly higher scores across all seven dimensions of the Critical Thinking Disposition Inventory compared to the control group (all *P* < 0.05). Specifically, the mean scores for the experimental group versus the control group were as follows: truth-seeking (51.36 ± 1.81 vs. 38.28 ± 1.54), open-mindedness (49.68 ± 1.52 vs. 34.96 ± 1.22), analyticity (54.68 ± 1.52 vs. 39.36 ± 1.60), systematicity (52.36 ± 1.90 vs. 36.36 ± 1.60), critical thinking confidence (51.16 ± 2.13 vs. 35.32 ± 1.62), curiosity (53.64 ± 1.55 vs. 38.36 ± 1.60), and cognitive maturity (48.48 ± 1.75 vs. 33.36 ± 1.60). The total critical thinking score was also significantly higher in the experimental group (361.36 ± 11.46) compared to the control group (256.96 ± 8.89; mean difference = 104.4, 95% CI: 95.1–113.7, *P* < 0.05).(outlined in Table 2; Fig. 6).

**Table 2:** Comparison of critical thinking dimensions between experimental and control groups

Assessment	Experimental Group (mean ± SD)	Control Group (mean ± SD)	*P*-value
Truth-seeking	51.360 ± 1.808	38.280 ± 1.537	< 0.05
Open-mindedness	49.680 ± 1.516	34.960 ± 1.216	< 0.05
Analyticity	54.680 ± 1.516	39.360 ± 1.597	< 0.05
Systematicity	52.360 ± 1.895	36.360 ± 1.597	< 0.05
Critical Thinking Confidence	51.160 ± 2.129	35.320 ± 1.618	< 0.05
Curiosity	53.640 ± 1.546	38.360 ± 1.597	< 0.05
Cognitive Maturity	48.480 ± 1.746	33.360 ± 1.597	< 0.05
Total	361.360 ± 11.464	256.960 ± 8.888	< 0.05

### Student evaluation of teaching methods

The experimental group rated the teaching methods significantly higher than the control group, with a mean score of 17.84 ± 1.12 versus 10.72 ± 1.59 (mean difference = 7.12, 95% CI: 5.51–8.73, *P* < 0.05). This indicates that students in the experimental group perceived greater effectiveness in knowledge acquisition, clinical reasoning, practical skills, self-directed learning, and teamwork abilities compared to those in the control group.

### Teaching satisfaction evaluation

**Fig. 7:**
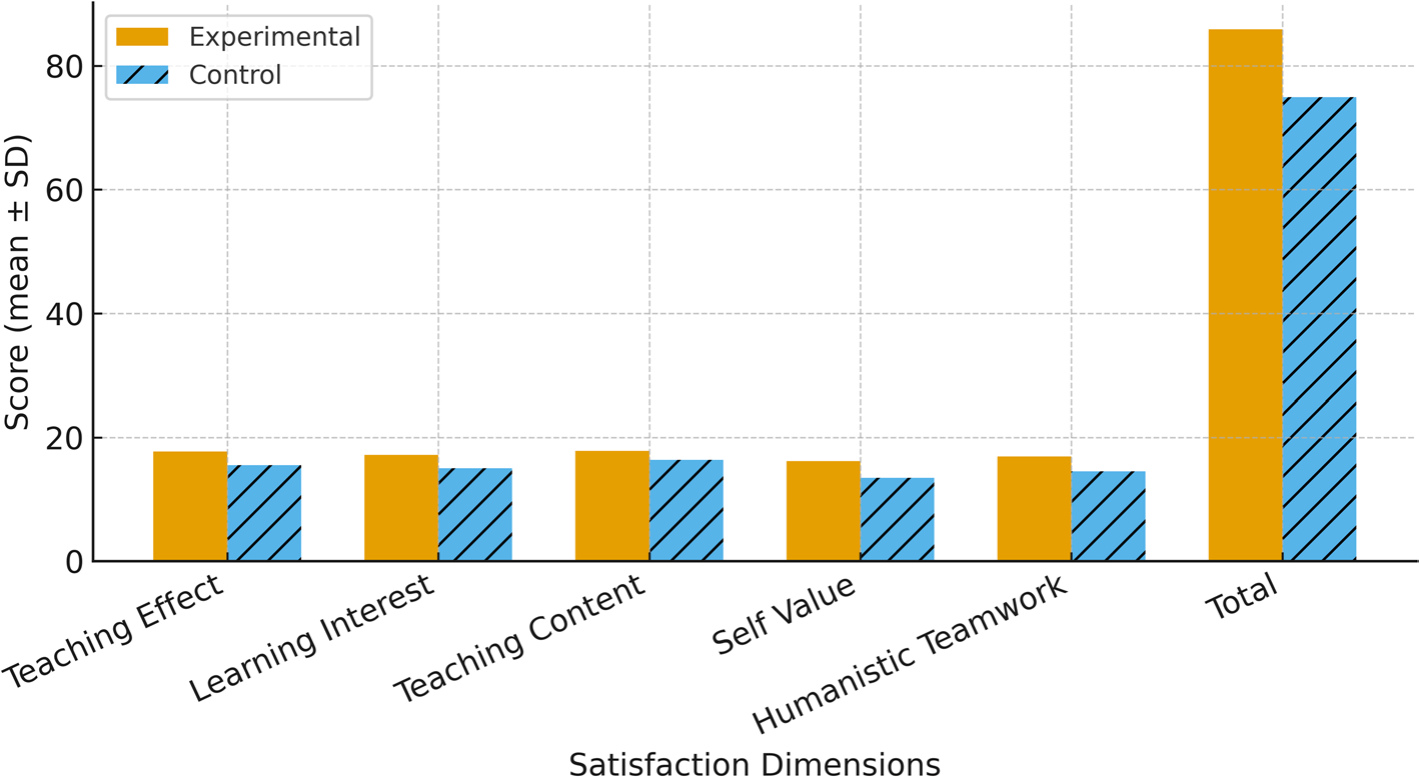
Comparison of teaching satisfaction scores between the experimental and control groups Data are presented as mean ± standard deviation across six dimensions of teaching satisfaction. Error bars represent 95% confidence intervals. Statistical differences between groups were assessed using independent-samples t-tests (*P* < 0.05)

The experimental group reported higher satisfaction across all six components of the teaching satisfaction questionnaire (all *P* < 0.05). Mean scores for the experimental group versus the control group were as follows: teaching effectiveness (17.76 ± 0.99 vs. 15.52 ± 1.36), learning interest (17.20 ± 0.98 vs. 15.04 ± 1.00), teaching content (17.84 ± 0.92 vs. 16.40 ± 1.17), self-worth (16.20 ± 1.06 vs. 13.48 ± 1.30), humanistic competence and teamwork (16.92 ± 0.89 vs. 14.52 ± 1.36). The overall teaching satisfaction score was higher in the experimental group (85.92 ± 4.34) compared to the control group (74.96 ± 5.64; mean difference = 10.96, 95% CI: 8.24–13.68,*P* < 0.05). The teaching satisfaction rate, defined as the proportion of students reporting “very satisfied” or “basically satisfied,” was also significantly higher in the experimental group, though exact percentages are not provided here as they were not specified in the data.(outlined in Table 3; Fig. 7).

**Table 3:** Comparison of teaching satisfaction between experimental and control groups

Assessment	Experimental Group (mean ± SD)	Control Group (mean ± SD)	*P*-value
Teaching Effect	17.760 ± 0.991	15.520 ± 1.360	< 0.05
Learning Interest	17.200 ± 0.980	15.040 ± 0.999	< 0.05
Teaching Content	17.840 ± 0.924	16.400 ± 1.166	< 0.05
Self Value	16.200 ± 1.058	13.480 ± 1.300	< 0.05
Humanistic Teamwork	16.920 ± 0.891	14.520 ± 1.360	< 0.05
Total	85.920 ± 4.344	74.960 ± 5.639	< 0.05

## Discussion

Trauma orthopaedics presents unique challenges in clinical education due to the complexity of fracture morphology, the intricacies of spatial anatomical structures, and the multifactorial nature of clinical decision-making. Traditional lecture-based teaching, which relies heavily on passive knowledge transmission and two-dimensional imaging, has proven insufficient in developing students’ spatial reasoning, critical thinking, and practical surgical skills. These limitations underscore the need for innovative teaching strategies that align with the demands of modern clinical education [23]. In this study, we integrated Problem-Based Learning (PBL) with three-dimensional visualization reconstruction technology (3D-VRT) into trauma orthopaedic education. The results demonstrated that this combined approach was associated with improved academic performance, critical thinking, and teaching satisfaction among medical students. Specifically, students in the experimental group outperformed those in the control group in written examinations (84.72 ± 2.39 vs. 79.52 ± 2.21, mean difference = 5.20, 95% CI: 3.62–6.78,*P* < 0.01) and clinical skills assessments (86.68 ± 2.22 vs. 79.24 ± 2.96, mean difference = 7.44,95% CI:5.82–9.06,*P* < 0.01), indicating enhanced theoretical understanding and practical competency.

PBL is a student-centered instructional method that promotes autonomous learning, clinical reasoning, and problem-solving in real-world contexts [24]. A substantial body of evidence has confirmed its effectiveness in enhancing learner motivation, deepening conceptual understanding, and fostering lifelong learning habits—benefits that are particularly pronounced in trauma orthopaedic education. Given the urgency, complexity, and spatial demands of trauma cases, students must be adept at rapid clinical judgment, anatomical interpretation, and surgical planning. PBL provides an immersive environment in which students actively explore diagnostic pathways and therapeutic strategies through simulated, yet clinically relevant, scenarios. Our findings indicate that PBL fosters the development of essential clinical competencies such as diagnostic acumen, interdisciplinary collaboration, and decision-making8.

The incorporation of 3D-VRT further strengthened this pedagogical model by overcoming the spatial limitations of traditional 2D imaging. By reconstructing high-resolution CT data into manipulable 3D models, students were able to rotate, zoom, section, and virtually reduce fracture sites, allowing for comprehensive multi-angle visualization of fracture patterns and surgical procedures [25]. This not only improved their spatial understanding but also facilitated mastery of complex anatomical relationships, fracture classifications, and operative strategies. Our findings are consistent with earlier research suggesting that 3D models enhance anatomical comprehension and support surgical training [26]. Students exposed to 3D-VRT demonstrated superior performance in both knowledge acquisition and skill execution [27], underscoring the added value of immersive, interactive technologies in orthopaedic education.Furthermore, the synergistic effect of combining PBL with 3D-VRT was evident in the development of students’ critical thinking abilities. Compared with the control group, students in the experimental group showed significantly higher scores in several dimensions of critical thinking, including truth-seeking, analytical reasoning, systematicity, and inquisitiveness. These cognitive attributes are essential for effective clinical practice, particularly in trauma scenarios where decision-making is often complex and time-sensitive. The iterative discussions inherent to PBL and the immersive spatial experiences provided by 3D models may have collectively contributed to the enhancement of these cognitive skills.

The intervention also improved student engagement and satisfaction. Higher ratings in teaching effectiveness (17.84 ± 1.12 vs. 10.72 ± 1.59, mean difference = 7.12, 95% CI: 5.51–8.73, *P* < 0.05) and overall satisfaction (85.92 ± 4.34 vs. 74.96 ± 5.64, mean difference = 10.96, 95% CI: 8.24–13.68,*P* < 0.05) were observed in the experimental group. These findings highlight the motivational and participatory benefits of the combined teaching strategy. The active learning environment fostered by PBL, together with the interactive nature of 3D visualization tools, enhanced students’ involvement, sense of agency, and practical relevance of the learning experience [28]. The ability to manipulate 3D fracture models and simulate surgical procedures likely increased both interest and perceived value of the learning content. These outcomes align with previous literature showing that interactive, learner-centered approaches improve satisfaction and intrinsic motivation. Within the PBL-3D model, educators adopt the role of facilitators rather than traditional lecturers, which supports student autonomy and self-confidence23. Student feedback further confirmed the perceived effectiveness of this model in fostering meaningful and engaging learning [29–31].By integrating self-directed learning with immersive virtual technologies into clinical case-based discussions, This study enhances the integration of theoretical concepts with real-world clinical training [32]. While objective assessments, including written and practical examinations, reflected enhanced learning performance, the higher satisfaction scores primarily represented students’ subjective perceptions of engagement and motivation.

Tools such as Mimics and 3D Slicer provided students with personalized and immersive learning experiences that are vital for mastering complex fracture patterns and operative techniques. Additionally, the use of PBL encouraged reflective thinking and independent problem-solving, both foundational to professional development. The observed improvements in teaching satisfaction suggest that students are more likely to remain engaged and retain knowledge—factors with potential long-term benefits for clinical practice. Importantly, under the PBL framework, instructors act as guides who support student autonomy while cultivating essential lifelong learning habits. These findings offer several implications for orthopaedic education: (1) Incorporating 3D-VRT into clinical teaching enhances anatomical visualization and supports procedural learning; (2) Embedding PBL in trauma orthopaedic education promotes contextualized and collaborative learning; and (3) Improvements in both objective (test performance, skills evaluation) and subjective (critical thinking, satisfaction) outcomes indicate that this hybrid teaching approach provides a comprehensive and effective strategy for undergraduate medical education in orthopaedics.

Nevertheless, this study has several limitations. First, it was conducted at a single institution with a relatively small sample size (*n* = 50), which may limit the generalizability of the findings. Future research should include larger, multi-center cohorts to validate these results across different educational contexts. Second, the study did not evaluate long-term knowledge retention or performance in real clinical settings, which are essential indicators of educational effectiveness. Longitudinal studies are warranted to assess the sustained impact of this combined approach on clinical competence and professional development.Third, a several confounding factors may have affected the findings, including differences in instructor teaching styles, the novelty of 3D visualization technology, and individual variations in student engagement. These factors should be considered when interpreting the results.Future studies should involve larger, multi-institutional samples and explore the integration of advanced technologies such as virtual reality (VR) or augmented reality (AR) in trauma orthopaedics education to further enhance immersive and interactive learning experiences.

## Conclusion

In summary, the integration of Problem-Based Learning (PBL) with Three-Dimensional Visualization Reconstruction Technology (3D-VRT) was associated with improved academic performance, critical thinking, and learning satisfaction among undergraduate medical students in trauma orthopaedics at our institution. These findings suggest that this combined teaching approach may serve as a valuable and feasible educational strategy in similar clinical teaching settings. However, given the single-institution and small-sample design, further multi-center studies are warranted to validate these results.

## Supplementary Information

Supplementary Material 1.

Supplementary Material 2.

## Data Availability

The datasets used and analyzed during the current study are available from the corresponding author on reasonable request.

## Abbreviations

PBL: Problem-Based Learning
CT: Computed tomography
3D-VRT: Three-dimensional visualization reconstruction technology
