# Supplementary material for: Application of problem-based learning combined with three-dimensional visualization reconstruction technology in trauma orthopedics teaching and its impact on teaching satisfaction
Source: BMC Med Educ. 2025 Dec 7;26:53. doi: 10.1186/s12909-025-08364-4 (PMC12797743; doi:10.1186/s12909-025-08364-4)
Supplement: Supplementary file 1 — Supplementary Material 1. [file 12909_2025_8364_MOESM1_ESM.doc]

**Orthopedic Teaching Evaluation Scale (Student Version)**

| **Evaluation Item** | **Scoring Criteria (Single Choice)** |
| --- | --- |
| 1. Knowledge acquisition | □1 point: Not at all □2 points: Somewhat □3 points: Noticeably □4 points: Very noticeably |
| (Understanding of trauma orthopedics theory) |  |
| 2. Development of clinical reasoning | □1 point: Not at all □2 points: Somewhat □3 points: Noticeably □4 points: Very noticeably |
| (Clinical analysis, critical thinking skills) |  |
| 3. Improvement of practical skills | □1 point: Not at all □2 points: Somewhat □3 points: Noticeably □4 points: Very noticeably |
| (e.g., demonstration of procedures, case management ability) |  |
| 4. Enhancement of self-directed learning | □1 point: Not at all □2 points: Somewhat □3 points: Noticeably □4 points: Very noticeably |
| (Enthusiasm for post-class independent inquiry) |  |
| 5. Teamwork abilities | □1 point: Not at all □2 points: Somewhat □3 points: Noticeably □4 points: Very noticeably |
| (Effectiveness of group discussions/collaborative learning) |  |

**Total Score Range**: 5-20 points (higher scores indicate better teaching effectiveness)

**Trauma Orthopedics Teaching Effectiveness Evaluation Questionnaire**

**Instructions**: Please mark “√” on the option that best reflects your true feelings for each item (1 point = Not at all, 2 points = Somewhat, 3 points = Noticeably, 4 points = Very noticeably).

A self-designed trauma orthopedics teaching evaluation questionnaire was distributed anonymously to both groups of students. The questionnaire included five items: *knowledge acquisition, development of clinical reasoning, improvement of practical skills, enhancement of self-directed learning, and teamwork abilities*. Each item was scored on a 4-point scale: Not at all (1 point), Somewhat (2 points), Noticeably (3 points), and Very noticeably (4 points), with a total score of 20 points. Higher scores indicate better teaching effectiveness.
