# Supplementary material for: Application of problem-based learning combined with three-dimensional visualization reconstruction technology in trauma orthopedics teaching and its impact on teaching satisfaction
Source: BMC Med Educ. 2025 Dec 7;26:53. doi: 10.1186/s12909-025-08364-4 (PMC12797743; doi:10.1186/s12909-025-08364-4)
Supplement: Supplementary file 2 — Supplementary Material 2. [file 12909_2025_8364_MOESM2_ESM.doc]

**Trauma Orthopaedics Teaching Satisfaction Evaluation Scale**

**Instructions**: Please mark “√” on the option that best reflects your true feelings for each item and write your score (0-20) in the provided space. Each domain is scored on a scale from 0 to 20, with higher scores indicating greater satisfaction. The total score is out of 100 points. Your responses will remain anonymous.

| **Domain** | **Scoring Criteria (0-20 points)** | **Your Score (0-20)** |
| --- | --- | --- |
| 1. Teaching Effectiveness | □ 0-5: Not satisfied □ 6-10: Somewhat satisfied □ 11-15: Moderately satisfied □ 16-20: Very satisfied (Evaluation of the instructor's ability to deliver clear and effective teaching) | _______ |
| 2. Learning Interest | □ 0-5: Not satisfied □ 6-10: Somewhat satisfied □ 11-15: Moderately satisfied □ 16-20: Very satisfied (Degree of interest and engagement in learning trauma orthopedics) | _______ |
| 3. Teaching content | □ 0-5: Not satisfied □ 6-10: Somewhat satisfied □ 11-15: Moderately satisfied □ 16-20: Very satisfied (Relevance, depth, and clarity of the teaching content) | _______ |
| 4. Self-Worth | □ 0-5: Not satisfied □ 6-10: Somewhat satisfied □ 11-15: Moderately satisfied □ 16-20: Very satisfied (Sense of personal growth and confidence gained from the course) | _______ |
| 5. Humanistic competence and teamwork | □ 0-5: Not satisfied □ 6-10: Somewhat satisfied □ 11-15: Moderately satisfied □ 16-20: Very satisfied (Development of empathy and patient-centered skills) | _______ |

**Total Score**: _______ (Sum of the five domain scores)

**Scoring Classification**:

- Total score ≥ 90: "Very satisfied"
- Total score 60–90: "Basically satisfied"
- Total score < 60: "Dissatisfied"

**Teaching Satisfaction Rate**: Calculated as (Number of students with "Very satisfied" + Number of students with "Basically satisfied") / Total number of students × 100%.

**Note**: Please provide your score (0-20) for each domain based on your experience. This questionnaire is self-developed and designed to evaluate your satisfaction with the trauma orthopedics teaching program. Ensure your total score is the sum of the individual domain scores.
